# *“I think taking herbal medicine first can help prevent*. *If it doesn’t work*, *then can take start taking the medication given by the doctors*.*”* Patients’ perceptions towards hypertension in Fiji

**DOI:** 10.1371/journal.pone.0285998

**Published:** 2023-08-28

**Authors:** Avock Johnny Jonathan, Masoud Mohammadnezhad, Filimone Raikanikoda

**Affiliations:** 1 Fiji Ministry of Health and Medical Services, Suva, Fiji Islands; 2 School of Nursing and Healthcare Leadership, University of Bradford, Bradford, West Yorkshire, United Kingdom; 3 Department of Health Education and Behavioral Sciences, Faculty of Public Health, Mahidol University, Nakhon Pathom, Thailand; 4 School of Public Health and Primary Care, Fiji National University, Suva, Fiji Islands; Federal Medical Centre, NIGERIA

## Abstract

**Background:**

Hypertension remains a public health challenge worldwide however, the prevention, detection, treatment and management of this condition are not highly prioritized. Health knowledge has an important impact on individual’s health. The ability to actively participate in screening, diagnosis and management of hypertension are influenced by patient’s knowledge of hypertension. To understand why hypertension is so difficult to control, it may be of benefit to gain an understanding of the patient’s perspective. Hence, the aim of the study is to explore the perceptions of patients on prevention and diagnosis of hypertension in Fiji.

**Methods:**

The study used a qualitative method approach. The study was conducted at the four purposively selected health centers in the Lautoka/ Yasawa medical subdivision. A purposive sampling was used which included all the patients who attended the SOPD, age more than 18 years and above, diagnosed with hypertension for 6 months or more and attended clinic at one of the 4 selected health centers. Semi-structured open-ended interview guide were used to collect data among patients through in-depth interviews. Thematic analysis was used manually to analyze the data using four steps that is immersion in the data, coding the data, creating categories and identifying themes / subthemes.

**Results:**

Twenty-five SOPD patients took part in the in-depth interview and the responses were grouped into two themes. The themes emerged included hypertension knowledge and diagnosis of hypertension in a closed family and self. Subthemes derived from the hypertension knowledge were measures of awareness, hypertension aetiology, risk perception, origin of information and concept of prevention. Sub themes derived from the diagnosis of hypertension in a closed family were perception when first diagnosed, hypertension in relation and hypertension impact. Patients’ knowledge on etiologies and risk factors of hypertension were generally poor. Majority of the participants learnt about hypertension in hospitals and few over radios and television. Diagnosis in a closed family triggered worrisome, fear and fright on some patients.

**Conclusion:**

Majority of the patients have less knowledge about various risk factors of hypertension. Worrisome, fearful, frightful, frustration and sadness were some of the reactions and emotions highlighted by the patients. It is important to design culturally tailored interventions that address the psychological and behavioral needs of the patients. Recommendation to conduct further studies to understand the perception of hypertension among the general public.

## Introduction

Hypertension is a worldwide epidemic affecting nearly one billion people and is the commonest risk factor for premature death and manageable chronic condition throughout the world. It is a serious medical condition that significantly increase the risk on target organs resulting in stroke, heart attack, blindness or renal failure [1, 2]. Globally, hypertension is estimated to affect 26% of the world’s population. According to a study by Albert, (2020), the prevalence is expected to increase to 29% by 2025 [3]. The prevalence of hypertension continues to increase in relation to economic growth, urbanization and lifestyle change. Worldwide, there is a significant increase in risk factors such as obesity, smoking and alcohol intake, inadequate fruits and vegetables diet and lack of exercise which contribute to high blood pressure [4].

Biological, social, and psychological factors are often considered as significant risks of hypertension. Psychological state of an individual greatly affects the physical condition of human body. The Health Belief Model (HBM) and the Protection Motivation Theory (PMT) are some of the examples that explain the association between perceptions of risk with health behavior. These theories emphasize that perception of risk is important in educating patients on their health-seeking behavior including preventive measures [5, 6].

Developed countries and neighbors to Fiji, such as Australia and New Zealand also report high cases of hypertension. In Australia, based on measured data from the 2017–2018 Australian Bureau of Statistics National Health Survey, about 1 in 3 people aged 18 years and above have high blood pressure. In New Zealand, high blood pressure affects 1 in 5 people. Steps 2013 study conducted in Samoa found 38% of the adults above 18 years are living with hypertension. In Fiji, hypertension is the second most common cause of heart disease. According to the latest World Health Organization (WHO) data published in 2018, hypertension deaths in Fiji reached 381 or 6.5% of total deaths. The age adjusted death rate is 59.07 per 100,000 of population that rank Fiji number 2 in the world [7, 8]. This issue needs to be addressed across all level and it should be every individual’s responsibility to prevent and control hypertension.

Individuals from different family background, cultural backgrounds, socioeconomic status, education background and age group have different perceptions of hypertension. Health knowledge has an important impact on individual’s health [9]. The ability to actively participate in screening, diagnosis and treatment of hypertension are influenced by patient’s knowledge of hypertension. To understand why hypertension is so difficult to control, it may be of benefit to gain an understanding of the patient’s perspective of their condition. Hence, the aim of the study is to explore the perceptions of SOPD patients on prevention and diagnosis of hypertension in Fiji.

## Methods

### Study design and setting

The study had applied a qualitative approach exploring the perceptions of hypertensive patients on hypertension knowledge and diagnosis of hypertension in a closed family and self in the Lautoka medical area from May 9^th^ to 19^th^, 2022. A qualitative study focuses on understanding people’s beliefs, experiences, attitudes, behavior and interactions towards health-related issues. It is also helpful in collecting genuine information from different socioeconomic demographics and provide valuable insights into public experiences [10].

The study was conducted at the four health centres in Lautoka Subdivision namely Lautoka, Punjas, Kamikamica Park and Viseisei Health centre. There are 8 health centres in Lautoka Subdivision however these four health centres were purposively selected because they usually run busy special outpatient department (SOPD) services. These health centres were noted to be the busiest centres in the Lautoka Medical area which cater for approximately 2000–3000 patients per week. These health centres run general outpatient services as well as SOPD for diabetes, hypertension, cardiac / dyslipidaemia. The function of these health centres is to promote wellness at primary health care level. The SOPD clinics in the 4 health centres are carried out twice a week (Tuesdays and Wednesdays). Approximately 250–300 patients attend SOPD clinic each week.

### Study sample

The study included all the hypertensive patients within the medical area and focused on essential hypertensive patients that attended SOPD clinics at the 4 primary health centres in Lautoka medical area in May, 2022.

The study sample (Hypertension patients) had fulfilled the following criteria: SOPD patients that attend SOPD clinics at the 4 selected health centres in Lautoka Subdivision during the period of data collection, diagnosed with hypertension for more than six months, age more than 18 years and above, of male or female gender, citizen of Fiji and lived in Lautoka and agreed to participate in the study.

The exclusion criteria included patients not willing to participate, those patients who attend SOPD clinics in other health facilities apart from the selected 4 health centres, patients with mental / psychiatric conditions (since they will not be able to accurately answer the questions as they may not be in a sound state of mind and patients with secondary hypertension or with diabetes (dual).

Purposive sampling was conducted among the patients who met the study criteria. Twenty-five SOPD patients were included in total, 6 participants from each health centre, who were approached using face to face in-depth interview until theoretical data saturation was achieved [11].

### Data collection tool

A semi structed interview guide was used to collect data. The questions were developed based on the literature review and in line with the study research questions. It had 2 sections with a total of 20 questions, the first section on demography had 7 questions followed by 13 open-ended questions for the second section. The instrument was piloted first at Punjas Health centre on the 9^th^ of May, 2022 which helped define research questions and was feasible reflected in the data collection. Two (2) interviews were conducted among patients and HCWs to see what their views were on the questions as well as the quality of interviews. The English version was used for both the patient and HCW.

### Study procedure and ethical consideration

After getting ethics approval, the Chief Medical Officer West (CMOW) and the Sub Divisional Medical Officer (SDMO) Lautoka were approached and formally informed about the study, Approval was granted by CMOW for the 4 health centres. The SOPD nurses at the 4 health centres were approached and informed about the study and their help was requested in locating SOPD patients by confirming their attendance for their SOPD clinic however they were not involved in the data collection process. Flyers that contain information about the research was placed at each SOPD clinics in the four health centres few days prior to the data collection.

A short introduction of the study was provided verbally (in English and Itaukei language) by the principal researcher (Avock Johnny Jonathan) and one research assistant (in Hindi language) to the patients while they waited for their turn for SOPD clinic consultation. The verbal introduction was carried out during the done for the 2 weeks of data collection. Together with the verbal introduction, information sheet was provided to all the SOPD patients in their preferred language. The patients who agreed to participate in the study at their own free time were given consent forms in their preferred language.

The patients who agreed to take part in the research were interviewed by the researcher in a quiet room at each health facility at the preferred time by both the participant and the researcher. Patients were interviewed in the SOPD room. Each interview lasted approximately 30–40 minutes. All interviews were recorded. The research assistant in the Hindustani language helped the researcher to translate during the interviews with 1 participant. In depth interviews were carried out until data saturation was achieved during the 2 weeks period of data collection.

### Data management and analysis

All interviews / discussions were transcribed verbatim by the principal researcher. Transcription was done on the same day of the interview. A review of transcriptions was done to correct errors and removed references of names and places ensuring anonymity for the participants. Data analysis was carried out after the transcriptions were clarified.

Thematic analysis was used manually to analyze the data in this study. Thematic analysis was carried out using four steps that is immersion in the data, coding the data, creating categories and identifying themes / subthemes [11–14]. The co-authors had read and re-read all interview / FGD transcripts and identified similar phrases and words for which number will be assigned. The coded data that had the similar characteristics were grouped together. Once grouping of similar data was completed, descriptive themes and sub–themes were identified to reflect the perception of patients and primary care workers.

### Study rigor

Study rigor refers to quality, authenticity, and truthfulness of findings of qualitative research relating to the degree of trust, or confidence, readers have in the results [15, 16]. They includes credibility, transferability, dependability and confirmability [17]. Specific strategies, related to these criteria can be used throughout the research process to increase the trustworthiness of qualitative work [18, 19]. These criteria and the associated strategies that were used in this study have been discussed in Table 1 below.

**Table 1:** Summary of criteria and strategies applied for the study rigor.

Criteria	Strategies
Accountability	A short introduction of the study was provided verbally by the researcher to all SOPD patients and primary HCWs on a weekly basis for the two weeks period. In-depth interviews and FGDs were conducted over the period of two weeks and each interview lasted for at least 30 minutes while each FGD lasted for at least 60 minutes. All in-depth interviews and FGDs were recorded and transcribed by the researcher on the same day. Every step of the thesis and results was discussed with the supervisors.
Dependability	This study was developed from the early stages through a systematic search of the existing literature. Same in—depth interview questions were asked to all SOPD patients while the FGD questions were same for all primary HCWs. Review of transcription was done to correct any error. All data was coded manually and all coding was checked and re-checked thoroughly.
Confirmability	Triangulation of data source was done by using two data collection tools, i.e., the in-depth semi structured interview and FGDs to collect data. Data was checked by the co-authors.
Transferability	Purposive sampling techniques was used in the research and In-depth interviews were carried out until data saturation was achieved.

## Results

Twenty-five SOPD patients had participated in the face-to-face interview. There were more female participants (68%) compared to male participants (32%). Majority of the participants were from 50 years to 60 years age group (68%). In terms of ethnicity, the participants were Itaukei (36%), Fijian of Indian Descent 60% and others (n = 1). Education level background, majority of the participants had studies up to secondary school level (68%). Each participant was assigned a number from Participant 1 to participant 25 (Table 2).

**Table 2:** Characteristics of SOPD patient participants (n = 25).

Characteristics		Frequency	Percentage
Gender	Male	8	32
	Female	17	68
Age groups	40–49 years	3	12
	50–59 years	9	36
	60–69 years	8	32
	70 years and above	5	20
Ethnicity	Itaukei	9	36
	Fijian of Indian Descent	15	60
Education level	No formal education	1	4
	Primary school education	2	8
	Secondary school education	17	68
	University education	5	20

### Themes and sub themes identified

From the thematic analysis, two major themes emerged which include knowledge on hypertension and diagnosis of hypertension in a close family/self. Each themes includes different sub-themes and categories (Table 3). Each participant was assigned a number from Participant 1 to participant 25. The gender and ethnicity of the participants are also indicated in the quotation; referenced as M for Male, F for Female and FOID for Fijian of Indian Descent.

**Table 3:** Themes, sub themes and categories of in-depth interview analysis.

Themes	Sub themes	Categories
**Hypertension knowledge**	Measure of awareness	Unaware of hypertension, Some information, A lot of information
	Hypertension aetiology	Hypertension in family, Stress, Unhealthy diet and food products, Smoking, Alcohol /kava, Unaware of the causes
	Risk perception	Men at more risk of hypertension, all are at risk of hypertension, older generation are at most risk, Middle age people are at most risk
	Origin of information	Hospital, Outreach clinic, Families, Television and radio, Mass Media and internet
	Concept of prevention	Cannot prevent hypertension, Diet controlled, no salt, no stress, Exercise, no to smoking /alcohol /kava, Herbal medicine
**Diagnosis of hypertension in a closed family and self**	Perception -when first diagnosed	Nervous, Angry, Fear, Sad, Worried
	Hypertension in relation	Hypertension in family, Hypertension in friends, Hypertension in communities
	Hypertension impact	Unaffected by hypertension diagnosis, Prompt checking and monitoring, Pandemic outbreak

#### Theme 1: Hypertension knowledge

The first few questions asked to SOPD patients were about their knowledge on hypertension. The patients have highlighted various aspects of hypertension information that they are aware of such as hypertension etiology, risk perceptions, origin of information and concept of prevention. These various aspects are highlighted in the sub themes below.

*Hypertension awareness*. A few patients had mentioned that they had no knowledge at all while most stated that they had a little bit of information about hypertension.

‘*I don’t know anything about high blood pressure*. *They only tell me I have high blood pressure*. *I think it’s too much pressure on the blood’* (P9, a 57-year-old FOID male).

Most of the participants had minimal information about hypertension.

*‘I know high blood pressure can cause stroke and heart attack if we don’t watch our diet*’ (P18, a 54-year-old Itaukei female).

Only two of the participants had most of the information’s about hypertension.

‘*I know hypertension arise due to our unhealthy lifestyle choice*, *from the unhealthy food we eat*, *salt intake*, *lack of rest and stress*, *smoking*, *alcohol*, *kava and lack of exercise*. *Also*, *it can cause stroke*, *heart attack and other heart problem’* (P1, a 67-year-old Rotuman male)

*Hypertension etiology*. Majority of the participants were able to identify at least two factors that can cause hypertension.

Majority have highlighted that stress is causing high blood pressure. Some of the reasons given by patients were stress from work place, stress looking after grandchildren, worried about financial support, worried about their children and their school welfare, worried about no job, worried about upcoming family function such as wedding, or even deaths in the family and pressure from the husband / partner.

*‘I think stressing too much and worrying a lot causes high blood pressure*. *For me*, *I used to work six days in a week*, *going to work in the morning and coming back in the afternoon as late as 5pm*, *to cook*, *clean and do the washing*. *This routine has caused high blood pressure*’ (P20, 63-year-old FOID female).

Another participant stated:

*‘It’s through unhealthy choices and not getting enough exercise*, *causing obesity and high blood pressure’* (P12, a 41-year-old Itaukei female)

Some participants stated smoking, alcohol, and kava can cause hypertension.

*‘High blood pressure can also be caused by smoking*, *it contains bad fats that can damage the heart and also alcohol and kava’* (P18, a 54-year-old Itaukei female).

Few participants believed that a person could get hypertension if its genetically linked.

*‘My dad had high blood pressure*, *and now I have high blood pressure*. *I think it runs in the family’* (P1, a 67-year-old Rotuman male)

Some of the participants also stated that unhealthy choice of diet is causing high blood pressure.

*‘Eating high amount of salt*, *oily food and meat every day can cause high blood pressure’* (P5, 60-year-old FOID female)

Few of the participants had mentioned that they don’t know the causes of hypertension.

*‘I don’t know anything about hypertension*. *I was only told when I came to the hospital that I had high blood pressure and I don’t know what’s causing it although I feel well and healthy’* (P24, a 69-year FOID female).

*Risk perception*. Majority of the participants stated that the middle aged and older generation are at more risk of developing hypertension.

*‘I believe the middle age and the older generation risking their life more because these are the age group where you actively working to feed your family*, *stress with finance*, *work*, *family and on the hand*, *we are the ones smoking*, *drinking alcohol*, *kava’* (P1, a 67-year-old Rotuman male)

Some of the participants had mentioned that men are more prone to hypertension because they tend to smoke, drink excessive alcohol and kava.

*‘In my opinion*, *men are at more risk than women because they drink kava nearly every night and smoke as well*. *After kava*, *something called washdown*. *They start drinking few bottles of beer and we have noticed that more men are dying in the village from the complication of hypertension’* (P17, a 51-year-old Itaukei female)

A few of the participants had mentioned that the older generation are at more risk because they do not exercise a lot and a few believe that it’s the age group whereby people easily develop high blood pressure.

*‘The older generation are at risk because they worry more, they also look after the whole family giving them more stress and they hardly do exercise’ (P8, a 66-year-old Itaukei female)*

Another participant stated:

*‘My dad had hypertension when he was old*, *around 50s and I also had hypertension in early 50s’* (P11, a 58-year-old FOID female).

A few participants have stated that the middle age group are at more risk of developing hypertension.

*‘I think the middle age group are at more risk because we now seeing them more in numbers attending SOPD clinic’* (P10, a 55-year-old FOID female).

A participant stated:

*‘The middle age group because they are smoking a lot*, *drinking kava and alcohol more nowadays’* (P2, 74-year-old Itaukei female)

*Origin of information*. Most of the SOPD patients have stated that they get information about hypertension from medical professionals when they visit the health centre.

*‘I was sick one time*, *I had continuous headache and came to the health centre*. *that’s when the doctor told me I had high blood pressure’* (P16, 55-year-old Itaukei female).

Some of the patients have stated that get information about hypertension from radios and TV.

*‘I was listening to a Hindi program and they were discussing about high blood pressure’* (P10, 55-year-old FOID female)

A participant had stated that she gets information about hypertension from community outreach clinic.

*‘The nurses will come around during village visit to check all our blood pressure and they always advise us when they see our blood pressure is high’* (P15, 77-year-old Itaukei male)

Another participant stated that she gets information about hypertension from church health screening.

*‘We have a health department in the church and they check our height*, *weight*, *BP and sugar every 6 months*. *From there I started to know about my high blood pressure’* (P12, 41-year-old Itaukei female)

Some participants stated that they get information about hypertension from the mass media and internet.

*‘I was watching a video about high blood pressure on Facebook and it was showing how high blood pressure can cause heart attack’* (P3, a72 year old Itaukei male)

A few participants stated that they get information about hypertension from their families and relatives.

*‘My family and relatives*. *Whenever someone die from hypertension in the family*, *we start talking about it and advising each other’* (P23, a 72-year-old FOID female)

*Concept of prevention*. A participant stated that hypertension cannot be prevented.

*‘Once hypertension is in the family*, *it cannot be prevented*. *Will live and die with it’* (P9, a 57-year-old FOID male)

While the rest of the participants agreed that blood pressure can be prevented.

*‘It’s a choice to live healthy or unhealthy*, *so yes*, *we can prevent blood pressure’* (P12, a 41-year-old Itaukei female).

A few participants stated by practicing a healthy proper diet.

*‘If we eat healthy*, *we will not be getting high blood pressure*. *We should eat more vegetables and fruits and cut down on meat*, *cholesterol and fast foods’* (P1, a 67-year-old Rotuman male)

A participant stated:

*‘We should not be eating a lot of salt in our food*, *especially raw salt’ (*P15, 77-year-old Itaukei male)

Some participants stated that avoiding stress can also prevent hypertension.

*‘We should not worry; we need a lot of rest*. *The more then tension and stress*, *the more our pressure will go up’* (P14, a 72-year-old FOID female)

Few patients stated that exercise can help prevent hypertension.

*‘By exercising a lot*, *we will burn more fats and cholesterol’* (P22, a 69-year-old FOID female)

Some participants also stated that smoking, drinking kava and alcohol should be stopped.

*‘More education and awareness to this young generation not to smoke and drink*. *Many are not aware of the consequences of drinking and smoking’* (P1, a 67-year-old Rotuman male)

A few participants stated that hypertension can be prevented by the use of herbal medicine.

*‘I think taking herbal medicine first can help prevent*. *If it doesn’t work*, *then can take start taking the medication given by the doctors*’ (P3, a 72-year-old Itaukei male)

#### Theme 2: Diagnosis of hypertension in a close family and self

Further questions were asked about the impact of hypertension on self and on other family members. Under this theme, it explored patients’ perception on themselves when they were first diagnosed and highlighted the impact it had on their family.

*Perceptions when first diagnosed*. Majority of the participants have stated that they have felt worried and unhappy about the news.

*‘When I first heard about it, I felt sad then I was worried about it. I didn’t want my children to know about it’ (P22, a 69-year-old FOID female)*

Some of the participants stated it made them feel bad and angry.

*‘I was in denial the first time I was told I had high blood pressure*. *That feeling of frustration kept coming on me and I wasn’t feeling good about the news’* (P15, a 77-year-old Itaukei male)

A participant stated:

‘*The first time I was told I had high blood pressure*, *I had fear of dying from heart attack and stroke’ (*P13, a 59-year-old FOID female)

Another participant stated:

‘*It gave me more stress and more high blood pressure’* (P18, a 54-year-old Itaukei female)

*Impact of hypertension in a relative*. Many of the participants stated that they knew people in the family, community, friends or relatives living with hypertension.

‘*My late father had died of high blood pressure and stroke*. *He wasn’t taking his medication every day*. *From the time he died*, *we usually visit the hospital regularly to get our blood pressure check’* (P21, a 47-year-old FOID female)

Another participant stated:

*‘I know a lot of people from the village that have high blood pressure too*. *We are all somehow related*. *Every time*, *the doctor and nurse come to the village*, *we always go together to get our blood pressure checked and get our medicine too’* (P2, 74-year-old Itaukei female)

A few of the participants stated that no one else in the family has high blood pressure.

‘*My husband and parents don’t have high blood pressure*. *I’m the only one at home living with high blood pressure but I also know my some of my uncles and aunties have high blood pressure too’* (P11, a 58-year-old FOID female)

*Hypertension impact*. Some of the participants have stated that hypertension in the family did not have any impact on them.

*‘My husband has high blood pressure too and he is still working while I carry out my normal routine at home*. *It didn’t really affect us because we taking good care of ourselves’* (P10, a 55-year-old FOID female)

Most of the patients have stated that it had affected them emotionally.

*‘I feel worried about it sometimes*. *What if one of us die soon*. *What will happen if my husband gets stroke or heart attack*. *I don’t want my children to have high blood pressure too’ (*P9, a 57-year-old FOID female)

Another participant stated:

*‘Cost of living is high and we live in town*. *Only my husband is working*. *The doctor has said for us to eat healthy food only*. *Buying vegetables and fruits every three*, *four days is expensive*. *Preparing healthy meals too is something I can’t afford every day’* (P17, a 51-year-old Itaukei female)

## Discussion

This study showed that the patients had low knowledge towards various risk factors of hypertension. They reflected negative emotional reactions to response their psychological needs. To answer the research questions, knowledge on hypertension, origin of information, etc were highlighted in the discussion.

### Hypertension knowledge

The growing number of hypertension cases in this current world is a worry for the nation and its health sector. While hypertension is prevalent in both high income and low- and middle-income countries (LMICs), although 80% of deaths due to cardiovascular diseases occur in LMICs [20]. This can be attributed to early detection which is easily achieved in developed countries with more facilities and awareness. However, in developing countries, the low hypertension knowledge and awareness together with late presentation contributing to higher mortality rate [20]. In this study, the participant who had no formal education had no knowledge about hypertension and those with tertiary education had better knowledge as compared to those with primary and secondary education. Majority of the participants said that they knew some information about hypertension and that it causes stroke and heart attack. However, a very few participants were able to state the causes and risks along with the complication that can occur. Findings from a study by Mohammed et al., (2020) that the odds of knowledge were higher among female, urban dwellers, higher level of education and young generation less than 50 years of age [21]. The results from this study were similar to literature findings as the more the education level, the better the knowledge on hypertension.

Most of the participants had different views on the risk perceptions. Majority had stated that the middle age and older generation are at more risk of developing hypertension. Based on gender, men are more likely to be affected than women. The association with gender based was more likely for men than women. Family history as stated by some participants as their reason why middle age and older generation are at more risk. Findings from the study conducted by Goldstein, et al., (2006) mentioned that men with one or two hypertensive parents had higher ambulatory BP than women with hypertensive parents, whereas offspring of normotensive parents exhibited no sex difference in BP [22].

Patients usually get information about hypertension through mass media and family discussions. A study conducted by Lin, et al., (2008) mentioned that traditional mass media (television, radio, newspapers and magazines) were still the most popular sources of information for hypertension knowledge [23]. In this present study, participants had different views as some stated that they get information about hypertension from radio and TV. Some had mentioned internet to be useful particularly Facebook and you tube. In Fiji, majority of the households have television and radios and with so many free to air channels, the public prefers television and radio as sources of information. However, in this study, majority of the participants have stated that through HCWs and hospital visitation, more information about hypertension were obtained. Findings from a study by Charitini (2012) also support that doctor remain the dominant information source while the media and magazines on health issues were reported more frequently than the family, pharmacy and internet [24, 25].

Prevention of high blood pressure has been recognized as the controlling key to hypertension risk factors and mortality rate especially in LMICs. Identification of the level of knowledge and attitude of the population is an optimum step to prevention [26]. Hypertension can be prevented by eliminating salt and fats from the diet and maintaining a regular exercise. Participants in this study also stated that blood pressure can be prevented through healthy lifestyle by exercising regularly, lowering salt, fat intake, avoiding smoking, alcohol, kava and stress.

Currently, approximately 75%–80% of the world’s population, including hypertensive patients, use herbal medicines due to the receptive nature of the body toward herbs, and the low incidence reported side effects [27]. A few of the participants have the belief that herbal medicine can prevent hypertension. A study by Hall, et al., (2021) had showed similar findings that belief in alternate traditional medicine by African American as a preference for natural remedy in the treatment of hypertension [28].

### Diagnosis of hypertension in a closed family and self

Hypertension in a close family member or friend also affects individual’s hypertension perceptions. It was noteworthy that majority of the patients in this study at least know someone in the family, relatives or a friend living with hypertension. However, a very few patients had stated that they did not know anyone else living with hypertension. For those patients living with another member of the family with hypertension; financial burden had been stated as an impact. To live healthy, requires a healthy choice of food which can be quite expensive and local home-grown food which is a disadvantage for urban dwellers patients hence not affordable. Furthermore, emotional stress has been reported by a few patients where they feel worried that their family members might end up with a sudden heart attack or stroke as they are non-compliant to medication [29]. However, a few patients had mentioned that living with another known hypertensive family member didn’t at all have an impact on themselves. A cross sectional study by Dilini, et al., showed that the prevalence of hypertension was significantly higher in those with a family history of hypertension [22], however the study did not show the impact of individuals in the family living with hypertension.

The impact on individuals’ feelings when newly diagnosed with hypertension has been described by the patients in this study as worrisome, fearful, frightful, frustrating and sadness. Many of these patients did not express their reasons. A few of the patients had mentioned anger and frustration as they were not ready to receive the bad news, and as they had felt fit and healthy all along. A study by Ian, et al., in 2019, concluded that despite the availability of new therapies, patients are still diagnosed late and experiencing both emotional and financial impacts from the disease [30]. Patients in this current study also had similar experiences, feeling emotional and financial impacts.

### Study limitation

The study was limited to SOPD patients and primary HCWs in Lautoka and findings may not be generalized to Fijis populations. Due to the setting of the study and the nature of the study participants, timing of data collection was limited to two weeks. Data was collected based on time available for the participants on their SOPD clinic days.

## Conclusion

Hypertension knowledge was low on patients. Patients with tertiary education had more knowledge than those who have primary and secondary education including those that never attended school. Risk perceptions differ among patients while majority had stated that the middle age and older generation are at most risk of developing hypertension. Genetics, obesity, smoking were some risk factors identified by some participants. Majority of the patients obtained their information about hypertension from hospitals and clinic. Many of the participants believed that hypertension can be prevented from living a healthy choice lifestyle and majority of the participants have highlighted salt reduction and avoidance of stress as the most common preventive measures. However, some participants still believe that herbal medicine can prevent and treat hypertension.

Emotional stress has affected majority of the participants in this study when they have learnt that another member of the family is living with hypertension. The fear of morbidity and mortality was high among patients. Worrisome, fearful, frightful, frustration and sadness were some of the reactions and emotions highlighted by the patients when they had first received the news from HCWs that they were newly or undiagnosed with hypertension. Future researchers are recommended to conduct studies to understand the perception of hypertension among the general public.
